# Economic Evaluation of a Web-Based Tailored Lifestyle Intervention for Adults: Findings Regarding Cost-Effectiveness and Cost-Utility From a Randomized Controlled Trial

**DOI:** 10.2196/jmir.3159

**Published:** 2014-03-20

**Authors:** Daniela N Schulz, Eline S Smit, Nicola E Stanczyk, Stef PJ Kremers, Hein de Vries, Silvia MAA Evers

**Affiliations:** ^1^CAPHRI School for Public Health and Primary CareDepartment of Health PromotionMaastricht UniversityMaastrichtNetherlands; ^2^Amsterdam School of Communication Research/ASCoRDepartment of Communication ScienceUniversity of AmsterdamAmsterdamNetherlands; ^3^Nutrition and Toxicology Research Institute Maastricht (NUTRIM)Department of Health PromotionMaastricht UniversityMaastrichtNetherlands; ^4^CAPHRI School for Public Health and Primary CareDepartment of Health Services ResearchMaastricht UniversityMaastrichtNetherlands

**Keywords:** randomized controlled trial, economic evaluation, cost-effectiveness, cost-utility, lifestyle behaviors, Internet interventions, Web-based, computer-tailoring

## Abstract

**Background:**

Different studies have reported the effectiveness of Web-based computer-tailored lifestyle interventions, but economic evaluations of these interventions are scarce.

**Objective:**

The objective was to assess the cost-effectiveness and cost-utility of a sequential and a simultaneous Web-based computer-tailored lifestyle intervention for adults compared to a control group.

**Methods:**

The economic evaluation, conducted from a societal perspective, was part of a 2-year randomized controlled trial including 3 study groups. All groups received personalized health risk appraisals based on the guidelines for physical activity, fruit intake, vegetable intake, alcohol consumption, and smoking. Additionally, respondents in the sequential condition received personal advice about one lifestyle behavior in the first year and a second behavior in the second year; respondents in the simultaneous condition received personal advice about all unhealthy behaviors in both years. During a period of 24 months, health care use, medication use, absenteeism from work, and quality of life (EQ-5D-3L) were assessed every 3 months using Web-based questionnaires. Demographics were assessed at baseline, and lifestyle behaviors were assessed at both baseline and after 24 months. Cost-effectiveness and cost-utility analyses were performed based on the outcome measures lifestyle factor (the number of guidelines respondents adhered to) and quality of life, respectively. We accounted for uncertainty by using bootstrapping techniques and sensitivity analyses.

**Results:**

A total of 1733 respondents were included in the analyses. From a willingness to pay of €4594 per additional guideline met, the sequential intervention (n=552) was likely to be the most cost-effective, whereas from a willingness to pay of €10,850, the simultaneous intervention (n=517) was likely to be most cost-effective. The control condition (n=664) appeared to be preferred with regard to quality of life.

**Conclusions:**

Both the sequential and the simultaneous lifestyle interventions were likely to be cost-effective when it concerned the lifestyle factor, whereas the control condition was when it concerned quality of life. However, there is no accepted cutoff point for the willingness to pay per gain in lifestyle behaviors, making it impossible to draw firm conclusions. Further economic evaluations of lifestyle interventions are needed.

**Trial Registration:**

Dutch Trial Register NTR2168; http://www.trialregister.nl/trialreg/admin/rctview.asp?TC=2168 (Archived by WebCite at http://www.webcitation.org/6MbUqttYB).

## Introduction

Noncommunicable chronic diseases are associated with various modifiable health risk behaviors, such as physical inactivity, bad nutrition, excessive drinking, and smoking [1]. An unhealthy lifestyle and the consequences involved are related to a reduced quality of life [2] as well as substantial health care costs [3,4]. Stimulating a healthy lifestyle is important to improve health and prevent illness and to also reduce health care costs, especially with current budget cuts in the Netherlands and other countries [5,6].

Computer tailoring can be used successfully as an intervention to promote behaviors associated with a healthy lifestyle [7]. When applying computer tailoring, personalized feedback is generated by a computer program based on an individual assessment [8]. Earlier studies have demonstrated that tailored information is perceived as more relevant than nontailored information [9]. Moreover, computer-tailored interventions have proven to be effective in stimulating a healthier lifestyle, such as smoking cessation [10], preventing smoking relapse [11], encouraging healthy nutrition [12], lowering alcohol intake [13], and increasing physical activity [14]. Previous research has also indicated that changing multiple lifestyle-related behaviors is likely to be more effective than changing only a single behavior [15]. A recent study has shown that tailored interventions that aim to reduce multiple health risk behaviors are not only successful in reducing unhealthy behaviors but also in simultaneously enhancing the overall well-being of the individual [16]. The delivery of computer-tailored interventions targeting multiple health risk behaviors through the Internet has various benefits: These programs can be applied in privacy and at a time and place the respondent finds convenient, many people can be reached at relatively low intervention cost because more than 90% of the Dutch population has Internet access nowadays [17], and because the system is computerized it can be easily combined with and/or integrated in other programs or interventions.

Some economic evaluations of Web-based and/or computer-tailored programs have been conducted to date (eg, [18-23]). In general, these studies have given a first indication that these interventions—most were single behavior change interventions—can indeed be cost-effective. To our knowledge, however, no economic evaluation of a Web-based computer-tailored intervention targeting multiple health risk behaviors has been conducted so far.

Web-based computer-tailored lifestyle interventions are an interesting and promising option to make the health care system more sustainable because of their proven clinical effectiveness and their potential cost-effectiveness due to relatively low intervention costs and wide reach. Thus, information regarding the cost-effectiveness of Web-based computer-tailored intervention programs is crucial for health care decision makers and the government in making evidence-based decisions regarding large-scale implementation of such programs [24]. The aim of the present study, therefore, is to assess from a societal perspective the cost-effectiveness and cost-utility of 2 different versions (sequential and simultaneous) of a Web-based computer-tailored lifestyle intervention for adults compared to a control group that received only a minimal intervention.

## Methods

### Study Design and Participants

The economic evaluation was embedded in a 2-year single-blind randomized controlled trial including 3 study groups. The study was approved by the Medical Ethics Committee of Maastricht University and the University Hospital Maastricht (MEC 09-3-016/NL27235.068.09) and registered by the Dutch Trial Register (NTR2168).

In October 2009, the Dutch Regional Health Authorities of North-Brabant and Zeeland conducted the quadrennial Adult Health Monitor 2009 among inhabitants of these 2 provinces. This questionnaire could be completed on paper or online via the Internet. Respondents who completed the online version of the questionnaire were invited to take part in the present study. The Adult Health Monitor was interconnected with and integrated into our Web-based lifestyle intervention. The study website was also open to the general public; therefore, it was also possible to register for participation in the trial directly on the study website without having completed the Adult Health Monitor.

**Figure 1:**
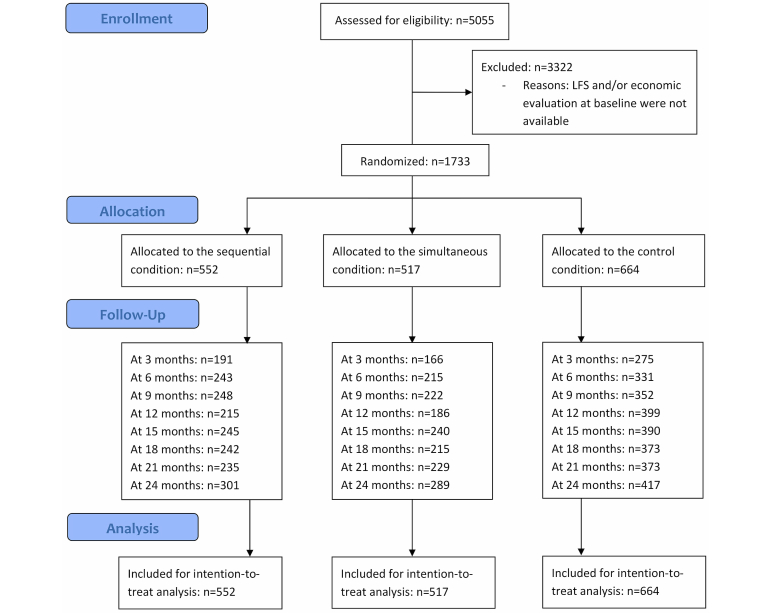
Flow diagram of the economic study.

The inclusion period for this study was from November 2009 up to and including July 2010. The following inclusion criteria were used: aged between 18 and 65 years, having a computer with Internet access and basic Internet literacy, and having a valid email address. Participants were randomized into 1 of 2 experimental groups (sequential condition or simultaneous condition) or into the control condition, with an equal probability of being assigned to any of the 3 groups. Randomization took place at the individual level by means of a computer software randomization system. Figure 1 shows the Consolidated Standards of Reporting Trials (CONSORT) flow diagram.

### Intervention

The intervention was a Web-based computer-tailored multisession program targeting adults. The primary aim of this lifestyle intervention was to motivate participants to be sufficiently physically active, to eat enough fruit and vegetables, to drink less alcohol, and to quit smoking. The intervention was based on the I-Change model, an integration of social cognitive models [25,26] and previously developed programs that have proven to be effective in increasing the level of physical activity [27], promoting the intake of fruit and vegetables [28], reducing the consumption of alcohol [29], and smoking cessation [30]. The respondents received feedback texts on their computer screens, which were aimed to motivate them to adopt the recommended health behaviors. All respondents received a health risk appraisal (HRA) indicating whether they adhered to the following public health guidelines: being moderately physically active for 30 minutes at least 5 days a week [31], eating 200 grams of vegetables per day [32], eating 2 pieces of fruit per day [32], not drinking more than 1 (for women) or 2 (for men) glasses of alcohol a day [32], and not smoking [33]. For all health risk behaviors, they received a traffic light image indicating whether they met (green), almost met (orange), or failed to meet (red) the guideline.

**Figure 2:**
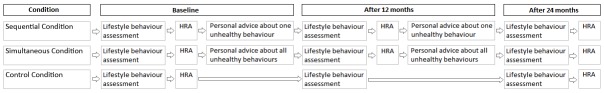
Design of the study. HRA: health risk appraisal.

The experimental groups subsequently received personalized advice provided in 4 steps based on questions about different psychosocial determinants of the I-Change model [25,26]: (1) attitude, (2) social influence, (3) preparatory planning, and (4) self-efficacy and coping planning. At the end of every step, personal advice was given to participants. At baseline, respondents in the sequential condition could select a module concerning one of the lifestyle behaviors for which they did not meet the public health guidelines (ie, received a red or orange traffic light in their HRA); on completing this module, they received personalized feedback regarding this particular behavior. After 12 months, a second assessment took place and respondents had the opportunity to choose a second module and to receive feedback on a second lifestyle behavior for which they did not meet the public health guidelines. At baseline and after 12 months, respondents in the simultaneous condition received feedback on all the behaviors they did not meet the public health guideline for at one time. In both conditions, an overview of all received pieces of advice was available (via a link which was also sent by email) for the respondent at the end of the sessions. The control group received the HRA at baseline and after 24 months, but did not receive any additional personal advice. Figure 2 presents the design of the study, including all parts of the intervention. A detailed description of the study protocol has been published elsewhere [34].

### Identification, Measurement, and Valuation of Costs and Effects

#### Overview

The economic evaluation was conducted from a societal perspective; therefore, all relevant costs (ie, intervention costs, health care costs, and respondent costs) and effects, such as quality-adjusted life years (QALYs) and lifestyle factor score (LFS), were taken into account [35].

#### Costs

Intervention costs consisted of hosting costs for the website, including costs for technical assistance and required updates. Costs for the development of the intervention program and research-specific costs were excluded because these are once-only costs that are not necessary again when implementing the program. The intervention costs were the same for all study groups because all groups received tailored advice that was integrated in the study website.

Health care costs included use of medication, medical consultations, inpatient and outpatient specialist care, hospital admissions, and other care (eg, professional home care). Health care costs were assessed using a 3-month retrospective questionnaire consisting of multiple choice and open-ended questions. This online questionnaire was taken quarterly during the 24 months. The updated Dutch Manual for Cost Analysis in Health Care was used to valuate costs [36]. If cost prices were not available, other sources were used. For instance, the website of the Healthcare Insurance Board [37] was used to calculate medication costs. The costs of medications were calculated based on the dose described by the respondent. Hence, costs per tablet, gram, or milliliter were used to calculate total medication costs for each respondent. Costs for health care services that could not be found in the Dutch Manual for Cost Analysis in Health Care were looked up on the Internet (eg, via the websites of health care services). If possible, 3 costs for each health care service were looked up to ultimately calculate a mean cost price for this service. Cost price details can be found in Multimedia Appendix 1.

Productivity costs included costs because of sickness absenteeism from work. They were calculated by the human capital method using mean costs for the Dutch population corrected for gender and age [36].

Respondent costs (also known as patient and family costs) included the time respondents spent on the website for participation and costs for traveling to health care services. For the time spent on the website, we used the mean time that was necessary to complete the program within the 3 study groups. The time lost due to participation in the Adult Health Monitor was also taken into account, but we only added the time people needed to fill out the parts of the Adult Health Monitor regarding the 5 lifestyle behaviors. We made this decision for 2 reasons: Firstly, it was not necessary to combine the interventions and the entire Monitor of the Regional Health Authorities; secondly, respondents who participated in the Adult Health Monitor skipped these parts on our website, whereas people who did not take part in the Adult Health Monitor completed these questions on our website (ie, in the end, all respondents completed the same number of questions). Ultimately, we used an average time of 70 minutes for the sequential condition, 100 minutes for the simultaneous condition, and 20 minutes for the control condition. To determine the cost of time spent on the website, we valued the time by applying the labor time using the mean costs of the Dutch population corrected for gender and age [36]. Costs for traveling to health care services were also valued in monetary terms. These costs were assessed based on average travel distances to health care services in the Netherlands [38] and the mean costs per kilometer [36].

#### Effects

For the cost-effectiveness analysis, the primary outcome measure was the total LFS. The following questionnaires were used to assess the 5 lifestyle behaviors: the Short Questionnaire to Assess Health-enhancing physical activity (SQUASH) [39,40], a 4-item Food Frequency Questionnaire (FFQ) assessing weekly fruit and fruit juice intake [39], a 4-item FFQ assessing the weekly consumption of boiled or baked vegetables as well as salads or raw vegetables [39], the 5-item Dutch Quantity-Frequency-Variability (QFV) questionnaire to assess alcohol intake [39,41], and questions asking participants if they smoked, what they smoked (cigarettes, cigars, or pipe tobacco), and how much they smoked per day (cigarettes) or per week (cigars or pipe tobacco) [39]. Based on the guidelines for physical activity, fruit intake, vegetable intake, alcohol consumption, and smoking, we calculated this LFS by summing all healthy behaviors (ie, complying with the guideline in question)—a similar method (Prudence score) was applied by Parekh et al [42]—at baseline and after 24 months; thus, the value of the LFS could range from 0 (adhering to no guidelines) to 5 (adhering to all guidelines). Moreover, a lifestyle factor change index (LFCI) was calculated by subtracting the LFS at baseline from the LFS at 24 months [43]. The value for this index could range from -5 to +5 on a continuous 10-point scale; positive scores indicated an increase, whereas negative scores indicated a decrease in the number of healthy behaviors.

For the cost-utility analysis, the primary outcome measure was utilities based on a health-related quality of life instrument. The Euro-Qol EQ-5D-3L questionnaire [44] was used to assess health-related quality of life and was completed by respondents every 3 months. The EQ-5D-3L consists of the following 5 health dimensions: mobility, self-care, daily activity, pain/discomfort, and anxiety/depression. On a 3-point Likert scale, respondents had to indicate their own state of health (no complaints=1; some complaints=2; many complaints=3). A utility score was calculated for each measurement point using the Dutch tariff [45]. This score could range from -0.33 (death) to 1 (perfect health). This utility score, in turn, was used to calculate the QALYs gained or lost during the 2-year study period by making use of the area under the curve method. The area under the curve stands for the duration of the health state (x-axis, 24 months) multiplied by the quality weight for the health state (y-axis, utility score).

#### Indexing and Discounting

The price year of this study was 2013. All cost prices were indexed to the year 2013 by using the consumer price indexes of 105.38 for the year 2009, 106.72 for the year 2010, 109.22 for the year 2011, 111.90 for the year 2012, and 115.00 for the year 2013 [46]. Because of the long-term follow-up of 2 years, costs made in the second year were discounted by 4%, and effects regarding the LFS assessed after 24 months and effects in QALYs measured in the second year of the study were discounted by 1.5% [36,47].

### Statistical Analyses

Respondents were included in the analyses when their LFS at baseline was available and when the economic evaluation measurement was completed at least at baseline.

To examine whether randomization was successful and whether the 3 groups were comparable in terms of demographics, baseline LFS, quality of life, and health care costs over the previous 3 months, analyses of variance (ANOVA) were used for continuous variables and chi-square tests for dichotomous or categorical variables. To investigate whether selective dropout had occurred, logistic regression analyses were used to compare (1) those who took part in the intervention but did not complete the questionnaires needed for the economic evaluation with those who did complete these questionnaires and were included in this study, and (2) of those included in this study, those who filled out at least 1 follow-up questionnaire regarding the economic evaluation with those who did not fill out any of the follow-up questionnaires.

Intention-to-treat analyses were performed. Mean imputation was used to fill in missing values regarding medication use, health care services, absenteeism from work, EQ-5D-3L items, and lifestyle items. When applying mean imputation, the mean of the previous and next value for the same variable was calculated. If mean imputation was impossible because of missing values on multiple measurement points, the last observation carried forward (LOCF) method was used to fill in missing values [48]. The next observation carried backward method was used when the value was not available on the baseline questionnaire. Unrealistic/impossible values (eg, more than 90 days absent from work in a period of 90 days) were recoded as the highest possible value. In case of unclear answers to the open-ended question regarding medication use (eg, private, too much, I do not know anymore), mean prices of the study group for this question were imputed.

To compare the 3 study groups regarding their biennial costs (ie, health care costs and respondent costs over a period of 2 years), nonparametric bootstrapping (5000 times) with 95% confidence intervals in percentiles was used. ANOVAs were performed to compare the groups regarding the LFS assessed after 24 months and the QALYs measured over the study period of 2 years.

Incremental costs (in Euros) and effects were calculated for all 3 study groups as well as a net monetary benefit by valuing the effectiveness and utility outcomes in monetary values using a threshold for society’s willingness to pay (WTP) per gain in the LFS (ie, per additional guideline met) and per QALY gained [49]. The probability of the highest net monetary benefit was presented from a WTP of €0 to €80,000 [50]. Additionally, we explicitly reported the probabilities when using a WTP of €18,000 because this is an accepted Dutch cutoff point per QALY gained as a result of preventive interventions [50].

### Uncertainty Analyses

For the cost-effectiveness and cost-utility analyses, bootstrapping resampling techniques (with 1000 times replacement) were carried out to deal with uncertainty around the estimates of cost-effectiveness and cost-utility. The results were presented in cost-effectiveness and cost-utility acceptability curves. Seven different sensitivity analyses were performed to deal with the uncertainty of parameter estimates from the primary analysis: (1) we executed the analyses from a health care perspective by excluding the productivity costs and the respondent costs (these might be reflected in participants’ reported quality of life anyway) [36]; (2) we excluded costs due to absenteeism from work because these costs differed significantly between the 3 study groups before the intervention; (3) we excluded respondents with less than 4 follow-up measurement points because of the large number of missing values (>50%); (4) we used a LFS change index as outcome variable to correct for the LFS before the intervention (cost-effectiveness analysis) and corrected the QALY for baseline utility (cost-utility analysis) [51]; (5) we excluded respondents with the highest costs based on the 95th percentile; (6) we did not discount costs and effect outcomes [36,47]; and (7) we discounted both the costs and effect outcomes by 4.0% instead of discounting only costs by 4.0% and effects by 1.5% [47].

Bootstrap analyses were done using Microsoft Office Excel 2010; all other analyses were done using SPSS version 20.0 (IBM Corp, Armonk, NY, USA).

## Results

### Sample Characteristics

A total of 1733 respondents were included in the analyses. Table 1 presents the baseline characteristics of the 3 study groups. One baseline difference was found between the groups: the sequential condition had significantly higher costs because of absenteeism from work compared to the control group.

**Table 1:** Comparability of the 3 study groups regarding demographics, health status, lifestyle behavior, and health care and travel costs over the past 3 months before baseline (N=1733)

Variable		Sequential (n=552)	Simultaneous (n=517)	Control (n=664)	*F* (*df*)	χ^2^ (*df*)	*P*
Age, mean (SD)		47.31 (11.62)	48.15 (11.96)	48.88 (12.19)	2.61 (2, 1730)		.07
**Gender, n (%)**							
	Men	279 (50.5)	264 (51.1)	366 (55.1)			
	Women	273 (49.5)	253 (48.9)	298 (44.9)		3.1 (2)	.21
**Educational level, n (%)**							
	Low	53 (9.9)	64 (12.9)	90 (13.7)			
	Medium	274 (51.3)	222 (44.7)	319 (48.7)			
	High	207 (38.8)	211 (42.5)	246 (37.6)		7.9 (4)	.10
**Income per month (€),** ^a^ **n (%)**							
	< 1,750	123 (22.9)	123 (24.7)	161 (24.5)			
	1750 - 3050	265 (49.3)	249 (50.0)	341 (52.0)			
	> 3050	150 (27.9)	126 (25.3)	154 (23.5)		3.2 (4)	.52
**Employment situation, n (%)**							
	Job (paid)	389 (72.2)	350 (70.3)	443 (67.4)			
	No job	150 (27.8)	148 (29.7)	214 (32.6)		3.2 (2)	.20
**Marital status, n (%)**							
	Relationship	423 (78.9)	402 (80.2)	507 (77.4)			
	Single	113 (21.1)	99 (19.8)	148 (22.6)		1.4 (2)	.50
Persons in household, mean (SD)		2.83 (1.65)	2.69 (1.24)	2.71 (1.37)	1.54 (2, 1695)		.21
**Native country, n (%)**							
	The Netherlands	511 (95.0)	480 (96.0)	627 (95.3)			
	Other	27 (5.0)	20 (4.0)	31 (4.7)		0.6 (2)	.73
BMI, mean (SD)		25.70 (4.18)	25.38 (3.91)	25.80 (5.71)	1.56 (2, 1714)		.21
Psychological distress, mean (SD)		44.46 (6.40)	45.00 (5.84)	44.74 (6.10)	1.00 (2, 1687)		.37
**Diseases,** ^b^ **n (%)**							
	Diabetes	25 (4.7)	20 (4.0)	23 (3.5)		1.0 (2)	.61
	Brain hemorrhage, TIA	2 (0.4)	4 (0.8)	7 (1.1)		1.9 (2)	.38
	Heart attack	8 (1.5)	5 (1.0)	5 (0.8)		1.5 (2)	.48
	Other serious heart disease	4 (0.7)	12 (2.4)	15 (2.3)		5.2 (2)	.07
	Cancer	12 (2.2)	7 (1.4)	11 (1.7)		1.1 (2)	.58
	High blood pressure	67 (12.5)	67 (13.4)	112 (17.2)		6.0 (2)	.05
	Asthma, COPD	36 (6.7)	34 (6.8)	43 (6.6)		0.01 (2)	.99
Lifestyle factor, mean (SD)		3.26 (1.04)	3.32 (1.07)	3.27 (1.06)	0.52 (2, 1730)		.59
Euroqol/utility (EQ-5D-3L), mean (SD)		0.89 (0.17)	0.89 (0.15)	0.90 (0.15)	0.40 (2, 1617)		.67
**Health care costs (€),** ^c^ **mean (SD)**							
	Medication use	44.62 (256.77)	39.14 (313.81)	31.68 (204.78)	0.39 (2, 1725)		.68
	Health care service	119.12 (401.90)	114.42 (201.50)	99.16 (184.52)	0.88 (2, 1729)		.42
	Admissions	32.95 (231.26)	31.98 (222.70)	62.85 (422.57)	1.88 (2, 1728)		.15
	Other care	34.04 (220.28)	21.28 (157.88)	40.41 (265.29)	1.08 (2, 1728)		.34
Absenteeism from work		676.68 (3135.31)	405.10 (2076.48)	328.19 (1413.07)	3.63 (2, 1674)		.03
Travel costs (€), mean (SD)		3.20 (10.42)	3.46 (6.41)	2.97 (5.63)	0.54 (2, 1579)		.59

^a^Respondents who did not want to report their income (n=257) were classified in the category €1751-€3050.

^b^TIA: transient ischemic attack; COPD: chronic obstructive pulmonary disease.

^c^Costs for prior 3 months.

### Dropout Analyses

Results from the logistic regression analysis showed that respondents who were excluded from the analyses of the present study (ie, those who took part in the intervention, but did not complete the questionnaires needed for the economic evaluation, n=3322) were significantly younger (beta=.03; *P*<.001), had a lower LFS (beta=–.12; *P*<.001), reported less diseases such as brain hemorrhage and transient ischemic attack (TIA) (beta=1.14; *P*=.04), and more of them did not have a paid job (beta=.40; *P*<.001) than respondents included in our analyses.

Each of the 8 retrospective questionnaires for the previous 3 months required for the economic evaluation was completed by at least 36.47% (632/1733) and not more than 57.76% (1001/1733) of the respondents, whereas 506 of 1733 respondents (29.20%) did not complete any of these questionnaires. The latter were younger (beta=.02; *P*=.002) and reported an unhealthier lifestyle (beta=–.12; *P*=.02) than those who completed at least 1 questionnaire.

After imputing missing values, total cost data was available for 1662 of 1733 respondents (95.90%), and effect data was available for all 1733 respondents (100%) for the LFS and for 1690 of 1733 respondents (97.52%) for QALYs.

### Costs and Effects

Table 2 shows that the simultaneous condition reported statistically significantly higher costs for health care services during the 2-year period than the control condition did. However, no differences were found regarding the total biennial health care costs. The travel costs were also statistically significantly higher for the simultaneous condition than the control condition. Because the simultaneous condition was the most time-intensive condition, followed by the sequential condition and then the control condition, total respondent costs were also higher among the simultaneous condition compared with the sequential and control conditions, and higher among the sequential condition compared to the control condition. Table 3 demonstrates that there were no differences found between the 3 study groups regarding effects on the LFS or on QALYs. Detailed information regarding the lifestyle scores at the different time points and effects of the intervention on the 5 behaviors are presented elsewhere [52].

**Table 2:** Mean biennial costs per participant per study group based on 5000 bootstrap replications.

Cost type		Costs per study group (€), mean (SD)			95% CI		
		Sequential	Simultaneous	Control	Sequential-Simultaneous	Control-Simultaneous	Sequential-Control
**Intervention costs**							
	Fixed hosting costs	0.54	0.54	0.54			
**Health care costs**							
	Medication use (n=1733)	193 (57)	200 (74)	162 (31)	-208.73, 166.37	-222.27, 91.00	-81.95, 175.03
	Health care service (n=1733)	719 (124)	861 (73)	641 (42)	-385.33, 169.11	-386.86, -56.07	-131.59, 368.62
	Admissions (n=1732)	396 (89)	303 (78)	461 (97)	-141.58, 325.48	-87.86, 401.07	-327.51, 200.20
	Other care (n=1732)	222 (67)	178 (40)	247 (67)	-97.69, 204.61	-76.51, 230.00	-211.27, 161.91
	Total health care costs (n=1714)	1521 (195)	1534 (162)	1502 (151)	-508.11, 504.92	-461.19, 399.62	-453.30, 493.35
**Productivity costs**							
	Absenteeism (n=1714)	3489 (652)	3563 (666)	2600 (538)	-1889.89, 1751.48	-2642.42, 673.86	-724.22, 2586.62
**Respondent costs**							
	Travel costs (n=1678)	25 (3)	30 (3)	24 (2)	-13.26, 3.42	-12.55, -0.41	-5.40, 9.10
	Fixed time costs (n=1733)	41 (0)	59 (1)	12 (0)	-18.65, -16.27	-47.71, -45.71	28.53, 29.95
	Total respondent costs (n=1678)	64 (3)	87 (3)	35 (2)	-30.66, -13.28	-57.81, -45.39	22.25, 37.19

**Table 3:** Mean biennial effect on the lifestyle factor score (LFS) and quality-adjusted life year (QALY) score per participant per study group.

Effect	Mean (SD) per study group			*F* (*df*)	*P*
	Sequential	Simultaneous	Control		
LFS^a^ (n=1733)	3.37 (1.08)	3.42 (1.06)	3.34 (1.05)	0.92 (2, 1730)	.40
QALY (EQ-5D-3L) (n=1690)	1.80 (0.30)	1.78 (0.31)	1.82 (0.27)	1.79 (2, 1687)	.17

^a^This factor could range from 0 (adherence to all guidelines) to 5 (adherence to no guideline at all), plus discounting effect.

### Cost-Effectiveness Analyses

Table 4 shows that the costs in the control condition were less than the costs in the sequential and the simultaneous conditions, but that the effects in the 2 experimental conditions were higher. From a WTP of €4594 or more, the sequential condition appeared more likely to be cost-effective than the control condition. From a WTP of €10,850 or more, the simultaneous condition seemed more likely to be cost-effective than the control condition. When comparing the cost-effectiveness of the 2 experimental conditions, the simultaneous condition seemed more likely to be cost-effective than the sequential condition from a WTP of €17,106.

**Figure 3:**
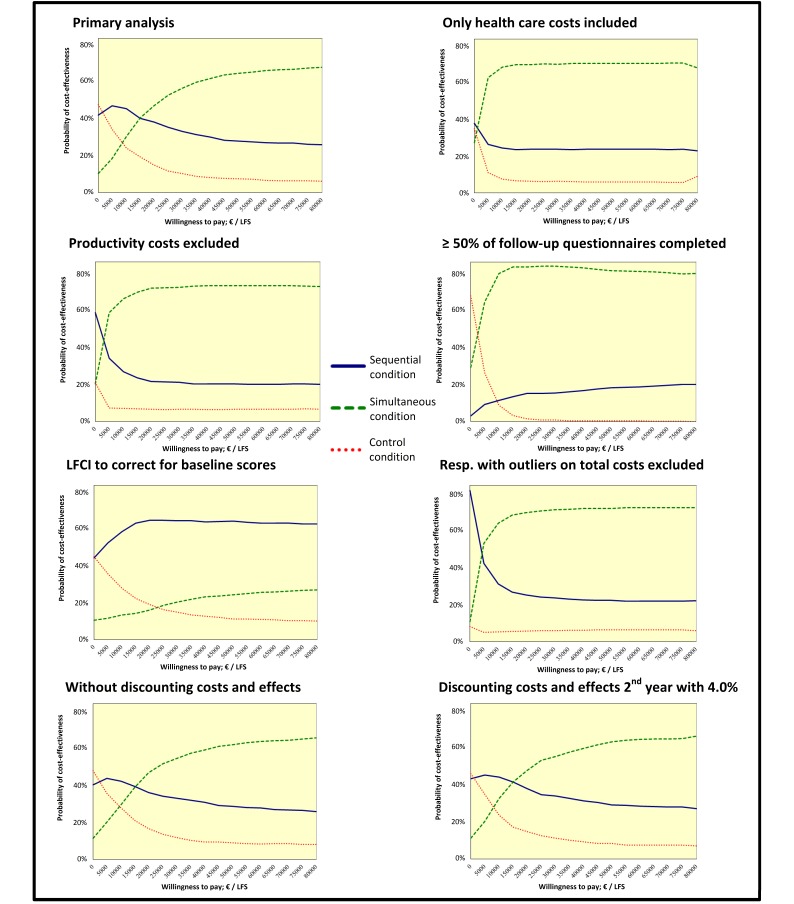
Cost-effectiveness acceptability curve for the study groups based on primary and sensitivity analyses.

The probability that the sequential condition was cost-effective at a WTP of €0 per gain in lifestyle score was 42%. For the simultaneous condition, the probability was 10% and for the control condition, the probability was 48% (see Table 5 and Figure 3). With a WTP of €18,000 per gain in lifestyle score, the simultaneous intervention would probably (ie, 45%) be the most cost-effective, followed by the sequential intervention (ie, 39%). The 2 sensitivity analyses performed with different discounts confirmed this finding. Results based on the other sensitivity analyses were slightly different. The sensitivity analysis using a LFCI as outcome variable (ie, correcting for the baseline lifestyle score) showed that the sequential condition might be most likely to be most cost-effective independent of the WTP. According to the sensitivity analysis performed from a health care perspective, the sensitivity analysis from which costs because of work absenteeism were excluded and the sensitivity analysis from which respondents with extremely high costs were excluded, both experimental conditions were found to be more likely to be cost-effective than the control condition regardless of the WTP. Based on the sensitivity analysis with inclusion of respondents who filled out the follow-up questionnaires at least 4 times (50%), the sequential condition was shown to be less likely to be cost-effective than the simultaneous and control condition for a WTP up to €9000.

**Table 4:** Incremental costs and effects per gain in lifestyle factor score (LFS) and per quality-adjusted life year (QALY) gained for the study groups with a willingness-to-pay threshold of €18,000.

Intervention		Incremental costs (€)^a^	Incremental effect^b^	Incremental costs per LFS/QALY (€)
**Lifestyle factor**				
	Sequential vs control	183.76	.04	4594.00
	Simultaneous vs control	868.00	.08	10,850.00
	Sequential vs simultaneous	–684.24	–.04	17,106.00
**QALY (EQ-5D-3L)**				
	Sequential vs control	183.76	–.01	Dominated^c^
	Simultaneous vs control	868.00	–.03	Dominated^c^
	Sequential vs simultaneous	–684.24	.02	Dominated^c^

^a^Costs per participant (€): sequential=4324.76; simultaneous=5009.00; control=4141.00.

^b^Lifestyle factor effects per participant: sequential=3.38, simultaneous=3.42, control=3.34; QALYs effects per participant: sequential=1.80, simultaneous=1.78, control=1.81.

^c^In one group, the costs are higher and the effects are lower compared to the other group.

**Table 5:** Results from cost-effectiveness and cost-utility analyses based on 1000 bootstrap replications for the sequential (seq), simultaneous (sim), and control (con) conditions.

Type of analysis		Study group (n)			Probability of highest net monetary benefit (%)								
		Seq	Sim	Con	WTP=€0			WTP=€18,000			WTP=€80,000		
					Seq	Sim	Con	Seq	Sim	Con	Seq	Sim	Con
**Primary analysis**													
	LFS	552	517	664	42	10	48	39	45	16	26	68	6
	QALY (EQ-5D-3L)	535	501	654	39	12	49	21	4	76	7	1	92
**Sensitivity analyses LFS**													
	Only health care costs included	552	517	664	38	27	35	24	70	6	24	71	6
	Productivity costs Excluded	552	517	664	59	20	21	22	71	7	20	73	7
	≥ 50% of follow-up questionnaires completed	100	104	195	3	29	68	14	83	2	20	80	0
	LFCI to correct for baseline scores	552	517	664	45	11	45	65	15	20	63	27	10
	Respondents with outliers on total costs excluded^a^	525	488	637	81	11	8	25	69	6	22	72	6
	Without discounting costs and effects	552	517	664	41	11	48	37	45	18	26	66	8
	Discounting costs and effects 2nd year with 4.0%	552	517	664	43	11	46	39	45	16	27	66	7
**Sensitivity analyses QALY (EQ-5D-3L)**													
	Only health care costs included	535	501	654	31	32	38	10	3	87	7	1	92
	Productivity costs excluded	535	501	654	56	19	25	14	2	84	7	1	93
	≥ 50% of follow-up questionnaires completed	100	104	195	6	25	70	5	21	74	7	21	73
	QALY corrected for baseline as outcome variable	513	482	621	38	18	43	36	13	51	29	8	63
	Respondents with outliers on total costs excluded^a^	509	475	627	80	9	11	25	3	73	13	2	85
	Without discounting costs and effects	535	501	654	38	12	50	22	4	75	8	1	91
	Discounting costs and effects 2nd year with 4.0%	535	501	654	40	12	48	22	4	75	7	1	92

^a^Based on the 95th percentile: €18,567.64.

### Cost-Utility Analyses

With regard to cost-utility, Table 4 shows that both the sequential and the simultaneous condition were dominated by the control condition. This means that the costs of the control condition were lower and the effects with regard to QALYs gained were higher in this group. When comparing the sequential condition with the simultaneous condition, costs were lower and effects on QALYs were larger in the sequential condition. Thus, the simultaneous condition was dominated by the sequential condition.

**Figure 4:**
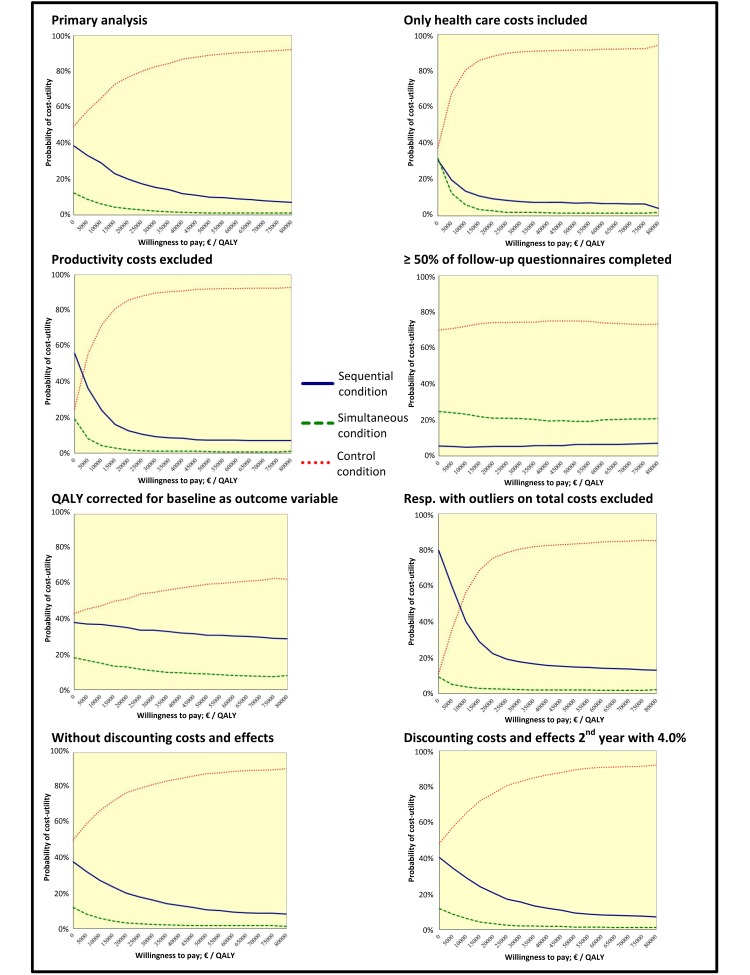
Cost-utility acceptability curves for the study groups based on primary and sensitivity analyses.

The probability that the sequential condition was most efficient at a WTP of €0 per QALY gained was 39%. For the simultaneous condition, the probability was 12% and for the control condition, the probability was 49% (see Table 5 and Figure 4). With a WTP of €18,000 per QALY gained, the cost-utility analysis showed that the control condition would probably (ie, 76%) be the most efficient condition. Indeed, Figure 4 shows that the control condition was most likely to be efficient, irrespective of the WTP. All but 3 sensitivity analyses confirmed these results. The sensitivity analysis in which costs due to work absenteeism were excluded and the sensitivity analysis in which respondents with extremely high costs were excluded showed that with a WTP up to €2980 and €7900, respectively, the sequential condition would probably be the most efficient (ie, >45% and >48% chance, respectively). The sensitivity analysis that included only those respondents who filled out the follow-up questionnaires at least 4 times (50% of all questionnaires) showed that the simultaneous condition was likely to be more efficient than the sequential condition, independent of the WTP. In all other analyses, this was the other way around.

## Discussion

### Principal Findings

An economic evaluation of 2 different versions of a Web-based computer-tailored multiple lifestyle intervention was performed. Despite some variations in the different sensitivity analyses outcomes, the results of this study give an indication that the 2 tailored intervention programs are likely to be more cost-effective when looking at lifestyles as a primary outcome than that of a control group, in which respondents received a short tailored overview. In general, the simultaneous intervention was likely to be most cost-effective, followed by the sequential intervention. However, the results were sensitive to baseline scores. When correcting for lifestyle behavior at baseline, the sequential intervention was probably most cost-effective. Regarding cost-utilities, the intervention received by the control group might be most preferable when compared to both lifestyle interventions (sequential and simultaneous).

With incremental costs of €4594 per gain in lifestyle score by meeting additional public health guidelines, the sequential condition is most likely to be cost-effective. With incremental costs of €17,106 or higher, the simultaneous version of the Web-based intervention is even more likely to be cost-effective than the sequential version. The incremental costs of our intervention seemed to be less favorable than the costs of €160 per guideline met in the study by Van Keulen et al [22]. However, the studies are hard to compare because the intervention tested by Van Keulen and colleagues [22] only considered 3 lifestyle behaviors (ie, physical activity, fruit intake, and vegetable intake) and tailored print communication was used instead of Web-based communication. Their study population consisted of older adults (aged 45-70 years). Most importantly, their control group received no intervention at all, whereas in our study the control group received a minimal intervention. Information regarding one’s lifestyle behavior may be sufficient to facilitate change and improve health risk behaviors [42]. Although the effects might be modest, in the present study the difference in effects between the control group who received the minimal intervention and the experimental conditions who received the sequential and simultaneous versions of the Web-based interventions might have become smaller. In a single behavior change intervention aimed at smoking cessation, Smit et al [21] reported incremental costs of €5100 per abstinent participant. This amount is comparable to our findings regarding gains in lifestyle behaviors, including smoking cessation. However, both studies [21,22] used shorter study periods (1.5 years and 1 year, respectively). This means that the cost-effectiveness regarding outcomes measured at the last follow-up in this study, which was after 2 years, cannot truly be compared with the cost-effectiveness results found in those studies.

Our finding that the control group might be most preferable regarding cost-utilities is comparable to the results reported by Smit et al [21] who found that the usual care their control group received was probably the most preferable intervention when compared with a Web-based computer-tailoring intervention and a combination of the Web-based intervention and face-to-face counseling by a practice nurse. A likely explanation for this finding may be that the follow-up period of 2 years was too short to find effects on quality of life. Although the intervention leads to lifestyle improvements that may prevent or postpone the incidence of a variety of lifestyle-related diseases, health gains from prevention programs often only become noticeable many years after the costs are made [53]. Moreover, baseline scores on the EQ-5D-3L were already high at the beginning of the study (mean 0.90, SD 0.15), which might be related to a restriction of this measurement tool (ie, the ceiling effect: the tool does not differentiate between high scores of the healthy utility range [54]). Consequently, the finding that our study population consisted of people reporting high scores on the EQ-5D-3L may be an explanation for not finding any statistically significant differences in QALYs gained between the groups. For public health interventions, it might be better to use other outcomes related to quality of life (eg, nonhealth outcomes, such as empowerment or satisfaction), which are more sensitive to changes in the short term because these quality of life measures tend to underestimate the relative benefits of this kind of intervention [55]. However, 2 cost-effectiveness acceptability curves (sensitivity analyses in which productivity costs were excluded and in which respondents with extremely high total costs were excluded) showed that the sequential condition might be preferable up to a WTP of €2700 and €8600, respectively, which is opposite to the findings by Van Keulen et al [22], whose control group was probably the most cost-effective intervention for ratios lower than €2851 per QALY gained.

In the literature, a WTP of €18,000 is the accepted cutoff point per QALY gained [50]. However, there is no such cutoff point regarding lifestyle changes as used in our study. This makes the interpretation of the results regarding cost-effectiveness complicated. Lifestyle interventions usually aim at preventing different kinds of diseases with different burdens. This makes it difficult to determine such a cutoff point, something that has also been pointed out for increases in smoking abstinence rates [21] or for each kilogram of body weight lost [56], for example. As suggested by Tate et al [24], for future research it would be good to transform the unit changes of different outcome measures into metrics that can be compared across different kinds of interventions (regardless of the target behavior). Thus, future research should aim to define a WTP cutoff point for different lifestyle behaviors or metrics that can be used for different lifestyle behaviors.

Furthermore, there has been discussion about the rates of discounting effects (eg, [36,57,58]), especially in the field of prevention. Effect outcomes should be discounted because the value of QALYs or health increases with time [59,60] and this value change is not taken into account in economic evaluations [61]. It has been argued that the same value of discounting should be used for costs and effects to be consistent [62], whereas it has been recommended that effects should be discounted at lower rates to correct for the increasing monetary value of health over time [36,47,53]. In our study, we reported the results without discounting, with 1.5% discounting, and with 4.0% discounting based on the guidelines for pharmacoeconomic research [47]. The similarity of the findings provides evidence for the robustness of our results.

The results revealed that respondents in the simultaneous condition reported higher costs because of health care service use during the 2-year study period than did respondents in the control condition. The travel costs in this group were also higher. These costs might be related to the number of visits to caregivers. The advice may have served as a kind of prompt among the respondents to ask caregivers for help in improving their lifestyle (eg, for smoking cessation guidance) [21]. However, it remains unclear why these costs were higher among respondents in the simultaneous intervention. It is conceivable that the marginal differences between the groups occurred because of measurement errors.

### Strengths and Limitations

To our knowledge, this was the first study assessing cost-effectiveness and cost-utility of 2 versions of a Web-based tailored lifestyle intervention aiming at multiple behavior change. Two outcome measures (ie, a LFS and QALYs) were used to evaluate costs and intervention effects over a relatively long period of 2 years. Seven different sensitivity analyses based on different views of economic evaluations found in the literature [36,51,57,62] were performed to test the robustness of the results. This is a further strong point of this study, although some analyses showed slightly different results.

There are some limitations that should be kept in mind when interpreting the results. First, we compared our intervention groups to a control group that also received a small amount of tailored information (ie, a personalized HRA). Because this HRA was integrated in the study website, the intervention costs were the same for all study groups. This strategy may have inflated the results and it may be that the cost-effectiveness would have been better if a different control group—who received either general information or no information at all—was used. Second, although we used a large sample (N=1733), the study suffered from high dropout rates, a common phenomenon in Web-based intervention studies (eg, [63-65]); therefore, many missing values for the follow-up assessments had to be imputed. Consequently, the imputation procedure may have distorted the reliability of the findings to some extent. That is, when using the conservative LOCF method to fill in the missing data for a large part of the sample, finding any intervention effects becomes more unlikely. The high dropout rates might have been caused by the need to assess health care costs and quality of life on a relatively large number of occasions. Although it might be good to measure health care use, medication use, absenteeism from work, and quality of life every 3 months to counteract recall bias [66], this may also be too time-consuming for some participants. This may have resulted in most of the respondents not completing all questionnaires and others dropping out of the intervention. Thus, future studies should aim to prevent loss to follow-up by sending tailored reminder emails [67], for example.

As presented in the dropout analysis, selective dropout occurred. Sensitivity analyses were performed to provide a more complete picture of the results among this selective group. Despite randomization of respondents to 1 of 3 study groups, statistically significant differences were found with regard to productivity costs, and some differences almost reached statistical significance (ie, age, high blood pressure, and serious heart diseases). These differences may have influenced the results. Also, we assessed absenteeism from work, but not “presenteeism” [68]. Finally, we used self-reported questionnaires that are subject to bias. Additional objective measures, such as medication registration at pharmacies and data from insurance companies, and cost diaries [69] could be included in future studies.

### Conclusions

Computer-tailored advice on lifestyle behaviors was likely to be cost-effective after 24 months when looking at lifestyles as a primary outcome. The Web-based tailored lifestyle intervention using a simultaneous approach is promising as the most cost-effective intervention in improving someone’s lifestyle, followed by the intervention using a sequential approach. However, with regard to improving quality of life, the control condition seemed to be preferable. Future studies should aim to compare different computer-tailoring conditions to a control group that does not receive any personalized advice, and to identify a cutoff point for the WTP for lifestyle changes.

## Multimedia Appendix 1

Cost prices for the different types of health care costs and absenteeism from work.

## Multimedia Appendix 2

CONSORT-EHEALTH checklist [70].

## Abbreviations

CONSORT: Consolidated Standards of Reporting Trials
FFQ: Food Frequency Questionnaire
HRA: health risk appraisal
LFCI: lifestyle factor change index
LFS: lifestyle factor score
LOCF: last observation carried forward
QALY: quality-adjusted life year
QFV: Quantity-Frequency-Variability
TIA: transient ischemic attack
WTP: willingness to pay
