# Supplementary material for: Economic Evaluation of a Web-Based Tailored Lifestyle Intervention for Adults: Findings Regarding Cost-Effectiveness and Cost-Utility From a Randomized Controlled Trial
Source: J Med Internet Res. 2014 Mar 20;16(3):e91. doi: 10.2196/jmir.3159 (PMC3978559; doi:10.2196/jmir.3159)
Supplement: Supplementary file 1 [file jmir_v16i3e91_app1.pdf]

Multimedia Appendix 1

Cost prices for the different types of health care costs and absenteeism from work

Health care and absenteeism from work	Price per visit (in €) [a]	Travel distance (in km) [b]
General practitioner or practice nurse (by phone, in the practice, at home)	30.92	1.1
Medical specialist (e.g. cardiologist, rheumatologist, internist, neurologist)	34.38	7.0
Alternative care giver (e.g. homeopath, acupuncturist)	55.33 [c]	2.2
Paramedical care giver (e.g. physiotherapist, ergo therapist, speech therapist)	33.40	2.2
Dietitian	29.46	2.2
Mental health care giver (e.g. psychologist, psychiatrist, psychotherapist)	83.37	5.0
Clinic for alcohol and drugs (CAD)	76.39 [c]	5.0
Other care givers	various [d]	various [d]
Hospital stay (per day)	551.10	7.0
Revalidation centre (per day)	371.04	7.0
Other paid care (e.g. professional home care; per hour)	38.20	n/a
Other unpaid care (from family, friends; per hour)	13.64	n/a
Sickness absenteeism from work (in days)	262.08 [e]	n/a
Medication [d]	various [f]	n/a

Note: [a] Dutch Manual for Cost Analysis in Health Care [36]; [b] costs per km: 0.20 € [36,38]; [c] mean price was calculated based on three prices, price details are available upon request; [d] price depended on the type of care giver, price details are available upon request; [e] calculated via the human capital method using mean costs for the Dutch population corrected for gender and age; [f] costs of medications were calculated based on the dose described by the respondent (per tablet, gram or ml), price was look up at www.medicijnkosten.nl, price details are available upon request.
