# Supplementary material for: Economic Evaluation of a Web-Based Tailored Lifestyle Intervention for Adults: Findings Regarding Cost-Effectiveness and Cost-Utility From a Randomized Controlled Trial
Source: J Med Internet Res. 2014 Mar 20;16(3):e91. doi: 10.2196/jmir.3159 (PMC3978559; doi:10.2196/jmir.3159)
Supplement: Supplementary file 2 [file jmir_v16i3e91_app2.pdf]

CONSORT-EHEALTH Checklist V1.6.2 Report

(based on CONSORT-EHEALTH V1.6), available at [http://tinyurl.com/consort-ehealth-v1-6].

3159

Date completed
12/3/2013 14:52:20
by
Daniela Schulz

Economic evaluation of a web-based tailored lifestyle intervention for adults: findings regarding cost-effectiveness and cost-utility from a randomized controlled trial

TITLE

1a-i) Identify the mode of delivery in the title
YES:
"[...] web-based tailored lifestyle intervention [...]"
1a-ii) Non-web-based components or important co-interventions in title
n/a
1a-iii) Primary condition or target group in the title
YES:
"[...] lifestyle intervention for adults [...]"

ABSTRACT

1b-i) Key features/functionalities/components of the intervention and comparator in the METHODS section of the ABSTRACT
"Methods. The economic evaluation, conducted from a societal perspective, was part of a 2-year randomized controlled trial including three study groups. All groups received personalized health risk appraisals based on the guidelines for physical activity, fruit intake, vegetable intake, alcohol consumption, and smoking. Additionally, respondents in the sequential condition received personal advice about one lifestyle behaviour in the first year and regarding a second behaviour in the second year; respondents in the simultaneous condition received personal advice about all unhealthy behaviours in both years. During a period of 24 months, health care use, medication use, absenteeism from work and quality of life (EQ-5D-3L) were assessed every three months using web-based questionnaires. Demographics were assessed at baseline, and lifestyle behaviours both at baseline and after 24 months. Cost-effectiveness and cost-utility analyses were performed based on the outcome measures lifestyle factor (the number of guidelines respondents adhered to) and quality of life, respectively. We accounted for uncertainty using bootstrapping techniques and sensitivity analyses."
1b-ii) Level of human involvement in the METHODS section of the ABSTRACT

1b-iii) Open vs. closed, web-based (self-assessment) vs. face-to-face assessments in the METHODS section of the ABSTRACT

1b-iv) RESULTS section in abstract must contain use data
"A total of 1,733 respondents were included in the analyses. From a willingness to pay of €4,594 per one additional guideline met, the sequential intervention (n = 552) was likely to be the most cost-effective, whereas from a willingness to pay of €10,850, the simultaneous intervention (n = 517) was likely to be most cost-effective. The control condition (n = 664), on the other hand, was probably preferred with regard to quality of life."
1b-v) CONCLUSIONS/DISCUSSION in abstract for negative trials
"Both the sequential and the simultaneous lifestyle interventions were likely to be cost-effective where it concerned the lifestyle factor, whereas the control condition was when it concerned quality of life. However, there is no accepted cut-off point for the willingness to pay per gain in lifestyle behaviours, making it impossible to draw firm conclusions. Further economic evaluations of lifestyle interventions are needed."

INTRODUCTION

2a-i) Problem and the type of system/solution
Our intervention is intended as a stand-alone intervention for adults of the general population; however, it was conntected to the Adult Health Monitor of different Regional Health Authorities in the Netherlands.

"Computer-tailoring can be used successfully as an intervention to promote behaviours associated with a healthy lifestyle [7]. When applying computer-tailoring, personalised feedback is generated by a computer programme based on an individual assessment [8]. Earlier studies have demonstrated that tailored information is perceived as more relevant than non-tailored information [9]. Moreover, computer-tailored interventions have proven to be effective in stimulating a healthier lifestyle, e.g. in reaching smoking cessation [10], preventing smoking relapse [11], encouraging healthy nutrition [12], lowering alcohol intake [13], and increasing physical activity [14]. Previous research has also indicated that changing multiple lifestyle related behaviours is likely to be more effective than changing only a single behaviour [15]. A recent study has shown that tailored interventions which aim to reducing multiple health risk behaviours are not only successful in reducing unhealthy behaviours but also in simultaneously enhancing the overall well-being of the individual [16]. The delivery of computer-tailored interventions targeting multiple health risk behaviours through the internet has various benefits: these programmes can be applied in privacy and at a time and place the respondent finds convenient; many people can be reached at relatively low intervention cost, since more than 90% of the Dutch population has internet access nowadays [17]; and since the system is computerized it can be easily combined with, and/or integrated in other programmes or interventions."
2a-ii) Scientific background, rationale: What is known about the (type of) system
"Some economic evaluations of web-based and/or computer-tailored programmes have been conducted to date [e.g., 18-23]. In general, these studies have given a first indication that these interventions – most were single behaviour change interventions – can indeed be cost-effective. To our knowledge, however, so far no economic evaluation of a web-based computer-tailored intervention targeting multiple health risk behaviours has been conducted.
Web-based computer-tailored lifestyle interventions are an interesting and promising option to make the healthcare system more sustainable, because of their proven clinical effectiveness and their potential cost-effectiveness due to relatively low intervention costs and wide reach."

METHODS

3a) CONSORT: Description of trial design (such as parallel, factorial) including allocation ratio
"The aim of the present study, therefore, is to assess from a societal perspective the cost-effectiveness and cost-utility of two different versions (sequential and simultaneous) of a web-based computer-tailored lifestyle intervention for adults, compared to a control group that received only a minimal intervention."
3b) CONSORT: Important changes to methods after trial commencement (such as eligibility criteria), with reasons
"Respondents were included in the analyses when their LFS at baseline was available and when the measurement regarding the economic evaluation was completed at least at baseline."
3b-i) Bug fixes, Downtimes, Content Changes
We sent additional (not planned) reminders in order to increase the response.
4a) CONSORT: Eligibility criteria for participants

"The following inclusion criteria were used: being between 18 and 65 years old; having a computer with internet access and basic internet literacy; and having a valid e-mail address."

4a-i) Computer / Internet literacy

"The following inclusion criteria were used: [...[basic internet literacy [...]."

4a-ii) Open vs. closed, web-based vs. face-to-face assessments:

"In October 2009, the Dutch Regional Health Authorities of North-Brabant and Zeeland conducted the quadrennial 'Adult Health Monitor 2009' among inhabitants of these two provinces. This questionnaire could be completed online via the internet or on paper. Respondents who completed the online version of the questionnaire were invited to take part in the present study. The Monitor was interconnected with and integrated into our web-based lifestyle intervention. The study website was also open to the general public, which means that it was also possible to register for participation in the trial directly on the study website without having completed the Monitor."

4a-iii) Information giving during recruitment

"Respondents who completed the online version of the questionnaire were invited to take part in the present study. For participation, they had to give informed consent and provide their e-mail address."

4b) CONSORT: Settings and locations where the data were collected

"In October 2009, the Dutch Regional Health Authorities of North-Brabant and Zeeland conducted the quadrennial 'Adult Health Monitor 2009' among inhabitants of these two provinces."

"The inclusion period for this study was from November 2009 up to and including July 2010."

4b-i) Report if outcomes were (self-)assessed through online questionnaires

"The intervention was a web-based computer-tailored multi-session programme targeting adults."

4b-ii) Report how institutional affiliations are displayed

5) CONSORT: Describe the interventions for each group with sufficient details to allow replication, including how and when they were actually administered

5-i) Mention names, credential, affiliations of the developers, sponsors, and owners

The study design is presented in the method section. Information is also given about the different study groups (sequential, simultaneous, control).

5-ii) Describe the history/development process

We had executed a pilot study in another province in the Netherlands (in Limburg).

5-iii) Revisions and updating

n/a - no major changes

5-iv) Quality assurance methods

5-v) Ensure replicability by publishing the source code, and/or providing screenshots/screen-capture video, and/or providing flowcharts of the algorithms used

5-vi) Digital preservation

5-vii) Access

Respondents of the Adult Health Monitor 2009 were allowed to take part in our study. However, the website was also open to the general public. Participation was free of charge and respondents were eligible to win prizes. For more information, see our study protocol: Schulz et al. Testing a Dutch web-based tailored lifestyle program among adults: a study protocol. BMC Public Health 2011;11:108)

5-viii) Mode of delivery, features/functionalities/components of the intervention and comparator, and the theoretical framework

"The intervention was a web-based computer-tailored multi-session programme targeting adults. The main aim of this lifestyle intervention was to motivate participants to be sufficiently physically active, to eat enough fruit and vegetables, to drink less alcohol and to quit smoking. All respondents received a health risk appraisal (HRA) indicating whether they adhered to the following public health guidelines: being moderately physically active for 30 minutes at least five days a week [25]; eating 200 grams of vegetables per day [26]; eating two pieces of fruit per day [26]; not drinking more than one (women) or two (men) glasses of alcohol a day [26]; and not smoking [27]. For all health risk behaviours, they received a traffic light indicating whether they met (green), almost met (orange) or failed to meet (red) the guideline. Subsequently, the experimental groups received personalized advice provided in four steps based on questions about different psychosocial determinants of the I-Change model [28]: (1) attitude; (2) social influence; (3) preparatory planning; and (4) self-efficacy and coping planning. At the end of every step, personal advice was given. At baseline, respondents in the sequential condition could select one module concerning one of the lifestyle behaviours for which they did not meet the public health guidelines and thus received a red or orange traffic light in their HRA; on completing this module, they received personalized feedback regarding this particular behaviour. After 12 months, a second assessment took place and respondents had the opportunity to choose a second module and to receive feedback on a second lifestyle behaviour for which they did not meet the public health guidelines. At baseline and after 12 months, respondents of the simultaneous condition received feedback on all behaviours for which they did not meet the public health guideline simultaneously. In both conditions, an overview of all received pieces of advice was available (via a link which was also sent by email) for the respondent at the end of the sessions. The control group received the HRA at baseline and after 24 months, but no additional personal advice. Figure 2 presents the design of the study, including all all parts of the intervention. A detailed description of the study protocol has been published elsewhere [29]."

5-ix) Describe use parameters

On the website, respondents could find information about the number of e-mails/invitations they will receive and about the duration of the study.

5-x) Clarify the level of human involvement

5-xi) Report any prompts/reminders used

see study protocol

5-xii) Describe any co-interventions (incl. training/support)

n/a - Our intervention was a stand-alone intervention, with a connection to the Adult Health Monitor of different Regional Health Authorities.

6a) CONSORT: Completely defined pre-specified primary and secondary outcome measures, including how and when they were assessed

The following (outcome) measures were included:

- demographic information
- health status
- lifestyle risk factor
- quality of life
- intervention costs
- health care costs
- productivity costs
- respondent costs

6a-i) Online questionnaires: describe if they were validated for online use and apply CHERRIES items to describe how the questionnaires were designed/deployed

6a-ii) Describe whether and how "use" (including intensity of use/dosage) was defined/measured/monitored

6a-iii) Describe whether, how, and when qualitative feedback from participants was obtained

6b) CONSORT: Any changes to trial outcomes after the trial commenced, with reasons

n/a - no changes

7a) CONSORT: How sample size was determined

7a-i) Describe whether and how expected attrition was taken into account when calculating the sample size

7b) CONSORT: When applicable, explanation of any interim analyses and stopping guidelines

n/a

8a) CONSORT: Method used to generate the random allocation sequence

"Participants were randomized into one of the two experimental groups (sequential condition (SeqC) or simultaneous condition (SimC)) or into the control condition (CC), with an equal probability of being assigned to any of the three groups. Randomization took place at the individual level by means of a computer software randomization system."

8b) CONSORT: Type of randomisation; details of any restriction (such as blocking and block size)

No block or cluster randomization was applied; the randomization was done at the individual level.

9) CONSORT: Mechanism used to implement the random allocation sequence (such as sequentially numbered containers), describing any steps taken to conceal the sequence until interventions were assigned

Also the control group got a small amount of personalized information in order to conceal allocation to the control group.

10) CONSORT: Who generated the random allocation sequence, who enrolled participants, and who assigned participants to interventions

Participants of the Adult Health Monitor were allowed to take part in our study. The computer-tailoring software, facilitated by the software-company, was used for the randomization process.

11a) CONSORT: Blinding - If done, who was blinded after assignment to interventions (for example, participants, care providers, those assessing outcomes) and how

11a-i) Specify who was blinded, and who wasn't

Our respondents were blinded.

11a-ii) Discuss e.g., whether participants knew which intervention was the "intervention of interest" and which one was the "comparator"

11b) CONSORT: If relevant, description of the similarity of interventions

n/a

12a) CONSORT: Statistical methods used to compare groups for primary and secondary outcomes

"Respondents were included in the analyses when their LFS at baseline was available and when the measurement regarding the economic evaluation was completed at least at baseline.
To examine whether randomization was successful and whether the three groups were comparable in terms of demographics, baseline LFS, quality of life and health care costs over the previous three months, analyses of variance (ANOVA) were used for continuous variables and Chi-square tests for dichotomous or categorical variables. To investigate whether selective drop-out had occurred, logistic regression analyses were used to compare (1) those who took part in the intervention, but did not complete the questionnaires needed for the economic evaluation, with those who did complete these questionnaires and were thus included in this study and (2) of those included in this study, those who filled out at least one follow-up questionnaire regarding the economic evaluation, with those who did not fill out any of the follow-up questionnaires.
Intention-to-treat analyses were performed. Mean imputation was used to fill in missing values regarding medication use, health care services, absenteeism from work, EQ-5D-3L items and lifestyle items. When applying mean imputation, the mean of the previous and next value for the same variable was calculated. In case mean imputation was impossible due to missing values on multiple measurement points, the last observation carried forward (LOCF) method was used to fill in missing values [40]. The next observation carried backward method was used when the value was not available on the baseline questionnaire. Unrealistic/impossible values (e.g. more than 90 days absent from work in a period of 90 days) were recoded into the highest possible value. In case of unclear answers to the open ended question regarding medication use (e.g., private, too much, I do not know anymore), mean prices of the study group for this question were imputed.
To compare the three study groups regarding their biennial costs (i.e., health care costs and respondent costs over a period of two years), nonparametric bootstrapping (5000 times) with 95% confidence intervals in percentiles was used. ANOVAs were performed to compare the groups regarding the LFS assessed after 24 months and the QALYs measured over the study period of two years.
Incremental costs (in Euros) and effects were calculated for all three study groups as well as a net monetary benefit by valuing the effectiveness and utility outcomes in monetary values using a threshold for society's willingness to pay (WTP) per gain in the LFS (i.e., per additional guideline met) and per QALY gained [41]. The probability of the highest net monetary benefit was presented from a WTP of €0 to a WTP of €80,000 [42]. Additionally, we explicitly reported the probabilities when using a WTP of €18,000 since this is an accepted Dutch cut-off point per QALY gained as a result of preventive interventions [42].

Uncertainty analyses
For the CEA and CUA, bootstrapping resampling techniques (with 1000 times replacement) were carried out to deal with uncertainty around the estimates of cost-effectiveness and cost-utility. The results were presented in cost-effectiveness and cost-utility acceptability curves. Seven different sensitivity analyses were performed to deal with the uncertainty of parameter estimates from the primary analysis: (1) we executed the analyses from a health care perspective by excluding the productivity costs and the respondent costs (these might be reflected in participants' reported quality of life anyway) [31]; (2) we excluded costs due to absenteeism from work because these costs differed significantly between the three study groups prior to the intervention; (3) we excluded respondents with less than four follow-up measurement points due to the large number of missing values (>50%); (4) we used a LFS change index as outcome variable to correct for the LFS prior to the intervention (cost-effectiveness analysis) and corrected the QALY for baseline utility (cost-utility analysis) [43]; (5) we excluded respondents with the highest costs based on the 95th percentile; (6) we did not discount costs and effect outcomes [31,39]; and (7) we discounted both the costs and effect outcomes by 4.0% instead of discounting only costs by 4.0% and effects by 1.5% [39].
Bootstrap analyses were done using Microsoft Office Excel 2010; all other analyses were done using SPSS version 20.0."

12a-i) Imputation techniques to deal with attrition / missing values

"Intention-to-treat analyses were performed. Mean imputation was used to fill in missing values regarding medication use, health care services, absenteeism from work, EQ-5D-3L items and lifestyle items. When applying mean imputation, the mean of the previous and next value for the same variable was calculated. In case mean imputation was impossible due to missing values on multiple measurement points, the last observation carried forward (LOCF) method was used to fill in missing values [40]. The next observation carried backward method was used when the value was not available on the baseline questionnaire. Unrealistic/impossible values (e.g. more than 90 days absent from work in a period of 90 days) were recoded into the highest possible value. In case of unclear answers to the open ended question regarding medication use (e.g., private, too much, I do not know anymore), mean prices of the study group for this question were imputed."

12b) CONSORT: Methods for additional analyses, such as subgroup analyses and adjusted analyses

Different sensitivity analyses were performed.

RESULTS

13a) CONSORT: For each group, the numbers of participants who were randomly assigned, received intended treatment, and were analysed for the primary outcome

We added a flow diagram of the economic study (see Figure 1).

13b) CONSORT: For each group, losses and exclusions after randomisation, together with reasons

See flow diagram. We excluded respondents in the case the lifestyle factor and/or the economic evaluation questionnaire was not available at baseline.

13b-i) Attrition diagram

YES: we added a flow/attrition diagram.

14a) CONSORT: Dates defining the periods of recruitment and follow-up

"The inclusion period for this study was from November 2009 up to and including July 2010."

14a-i) Indicate if critical "secular events" fell into the study period

14b) CONSORT: Why the trial ended or was stopped (early)

n/a

15) CONSORT: A table showing baseline demographic and clinical characteristics for each group

YES: see Table 1.

15-i) Report demographics associated with digital divide issues

YES: see Table 1.

16a) CONSORT: For each group, number of participants (denominator) included in each analysis and whether the analysis was by original assigned groups

16-i) Report multiple "denominators" and provide definitions

See Figure 1 (flow diagram)

16-ii) Primary analysis should be intent-to-treat

Yes, we reported ITT-analyses and also performed different sensitivity analyses.

17a) CONSORT: For each primary and secondary outcome, results for each group, and the estimated effect size and its precision (such as 95% confidence interval)

See Tables 2, 3, 4 and 5

17a-i) Presentation of process outcomes such as metrics of use and intensity of use

17b) CONSORT: For binary outcomes, presentation of both absolute and relative effect sizes is recommended
n/a
18) CONSORT: Results of any other analyses performed, including subgroup analyses and adjusted analyses, distinguishing pre-specified from exploratory
See results section
18-i) Subgroup analysis of comparing only users

19) CONSORT: All important harms or unintended effects in each group
No intended harm were reported.
19-i) Include privacy breaches, technical problems
It might be that technical problems occurred (in all study groups).
19-ii) Include qualitative feedback from participants or observations from staff/researchers

DISCUSSION

20) CONSORT: Trial limitations, addressing sources of potential bias, imprecision, multiplicity of analyses
20-i) Typical limitations in ehealth trials
"There are some limitations that should be kept in mind when interpreting the results however. First, we compared our intervention groups to a control group which also received a small amount of tailored information (i.e. a personalized HRA). As this HRA was integrated in the study website, this means that the intervention costs were the same for all study groups. This strategy may have inflated the results, and it may be that cost-effectiveness would have been better if a different control group – who received either general information or no information at all – was used. Second, although we used a large sample (n = 1,733), the study suffered from high drop-out rates – a common phenomenon in web-based intervention studies [e.g. 54-56] – which means that many missing values for the follow-up assessments had to be imputed. Consequently, the imputation procedure may have distorted the reliability of the findings to some extent. That is, when using the conservative LOCF-method to fill in the missing data for a great part of the sample, finding any intervention effects becomes more unlikely. The high drop-out rates might have been caused by the need to assess health care costs and quality of life on a relatively large number of occasions. While it might be good to measure health care use, medication use, absenteeism from work and quality of life every three months to counteract recall bias [57], this may also be (too) time-consuming for some participants. This may have resulted in most of the respondents not completing all questionnaires and others dropping out of the intervention. Future studies should thus aim to prevent loss to follow-up, for example by sending tailored reminder e-mails [58]. As presented in the dropout analysis, selective drop-out occurred. Sensitivity analyses were performed to provide a more complete picture of the results among this selective group. Third, despite randomization of respondents to one of the three study groups, statistically significant differences were found with regard to productivity costs; and some differences almost reached statistical significance (i.e., age, high blood pressure and serious heart diseases). These differences may have influenced the results. Finally, we used self-reported questionnaires which are subject to bias. Additional objective measures, such as medication registration at pharmacies and data from insurance companies, and cost-diaries [59] could be included in future studies."
21) CONSORT: Generalisability (external validity, applicability) of the trial findings
21-i) Generalizability to other populations
"As presented in the dropout analysis, selective drop-out occurred. Sensitivity analyses were performed to provide a more complete picture of the results among this selective group."
21-ii) Discuss if there were elements in the RCT that would be different in a routine application setting

22) CONSORT: Interpretation consistent with results, balancing benefits and harms, and considering other relevant evidence
22-i) Restate study questions and summarize the answers suggested by the data, starting with primary outcomes and process outcomes (use)

"An economic evaluation of two different versions of a web-based computer-tailored multiple lifestyle intervention was performed. The results of this study give an indication that the two tailored intervention programmes are likely to be more cost-effective when looking at lifestyles as a primary outcome, than that of a control group, in which respondents received a short tailored overview. In general, the simultaneous intervention was likely to be most cost-effective, followed by the sequential intervention. However, the results were sensitive to baseline scores. When correcting for lifestyle behaviour at baseline, the sequential intervention was probably most cost-effective. Regarding cost-utilities, the intervention received by the control group might be most preferable when compared to both lifestyle interventions (sequential and simultaneous).
With incremental costs of €4,594 per gain in lifestyle score by meeting additional public health guidelines, the sequential condition is most likely to be cost-effective; with incremental costs of €17,106 or higher, the simultaneous version of the web-based intervention is even more likely to be cost-effective than the sequential version. The incremental costs of our intervention seemed to be less favourable than the costs of €160 per guideline met in the study by Van Keulen et al. [22]. However, the studies are hard to compare, as the intervention tested by Van Keulen and colleagues [22] only considered three lifestyle behaviours (i.e., physical activity, fruit intake and vegetable intake), and tailored print communication was used instead of web-based communication. Their study population consisted of older adults (45-70 years). Most importantly, their control group received no intervention at all whereas in our study the control group received a minimal intervention. Information regarding one's lifestyle behaviour may be sufficient to facilitate change and improve health risk behaviours [34]. Though the effects might be modest, in the present study the difference in effects between the control group who received the minimal intervention and the experimental conditions who received the sequential and simultaneous versions of the web-based interventions might have become smaller. In a single behaviour change intervention aiming at smoking cessation, Smit et al. [21] reported incremental costs of €5,100 per abstinent participant. This amount is comparable to our findings regarding gains in lifestyle behaviours, including smoking cessation. Both studies [21,22], however, used shorter study periods (1.5 years and 1 year, respectively). This means that the cost-effectiveness regarding outcomes measured at the last follow-up in this study, which was after two years, cannot truly be compared with the cost-effectiveness results found in those studies.
Our finding that the control group might be most preferable regarding cost-utilities is comparable to the results reported by Smit et al. [21] who found that the usual care their control group received was probably the most preferable intervention when compared to a web-based computer-tailoring intervention and a combination of the web-based intervention and face-to-face counselling by a practice nurse. A likely explanation for this finding may be that the follow-up period of two years was too short to find effects on quality of life. Health gains from prevention programmes often only become noticeable many years after the costs are made [44]. Moreover, baseline scores on the EQ-5D-3L were already high at the beginning of the study (mean = .90; SD = .15), which might be related to one restriction of this measurement tool, i.e. the ceiling effect: the tool does not differentiate between high scores of the healthy utility range [45]. Consequently, the finding that our study population consisted of people reporting high scores on the EQ-5D-3L may be an explanation for not finding any statistically significant differences in QALYs gained between the groups. For public health interventions, it might be better to use other outcomes related to quality of life (e.g. non-health outcomes, such as empowerment or satisfaction), which are more sensitive to changes in the short term since these quality of life measures tend to underestimate the relative benefits of this kind of intervention [46]. However, two cost-effectiveness acceptability curves (sensitivity analyses in which productivity costs were excluded, and in which respondents with extremely high total costs were excluded) showed that the sequential condition might be preferable up to a WTP of €2,700 and €8,600 respectively, which is opposite to the findings by Van Keulen et al. [22], whose control group was probably the most cost-effective intervention for ratios lower than €2,851 per QALY gained.
In the literature, a WTP of €18,000 is the accepted cut-off point per QALY gained [42]. However, there is no such cut-off point regarding lifestyle changes as used in our study. This makes the interpretation of the results regarding cost-effectiveness complicated. Lifestyle interventions usually aim at preventing different kinds of diseases with different burdens. This makes it difficult to determine such a cut-off point, something which has also been pointed out, for example, for increases in smoking abstinence rates [21] or for each kg of body weight lost [47]. As suggested by Tate et al. [24], for future research, it would be good to transform the unit changes of different outcome measures into metrics that can be compared across different kinds of interventions (regardless of the target behaviour). Thus, future research should aim to define a willingness to pay cut-off point for different lifestyle behaviours or metrics which can be used for different lifestyle behaviours.
Furthermore, there has been discussion about the rates of discounting effects [e.g. 31,48,49], especially in the field of prevention. Effect outcomes should be discounted because the value of QALYs or health increases with time [50,51] and otherwise this value change is not taken into account in economic evaluations [52]. On the one hand, it has been argued that the same value of discounting should be used for costs and effects in order to be consistent [53], while on the other hand it has been recommended that effects should be discounted at lower rates to correct for the increasing monetary value of health over time [31,39,44]. In our study, we reported the results without discounting, with 1.5% discounting and with 4.0% discounting, based on the guidelines for pharmacoeconomic research [39]. The similarity of the findings provides evidence for the robustness of our results.
The results revealed that respondents in the simultaneous condition reported higher costs due to health care service use during the study period of two years than did respondents in the control condition. The travel costs in this group were also higher. These costs might be related to the number of visits to care givers. The advice may have served as a kind of prompt among the respondents to ask caregivers for help in improving their lifestyle, e.g. for smoking cessation guidance [21]. However, it remains unclear why these costs were higher among respondents in the simultaneous intervention."

22-ii) Highlight unanswered new questions, suggest future research

- "As suggested by Tate et al. [24], for future research, it would be good to transform the unit changes of different outcome measures into metrics that can be compared across different kinds of interventions (regardless of the target behaviour). Thus, future research should aim to define a willingness to pay cut-off point for different lifestyle behaviours or metrics which can be used for different lifestyle behaviours. [...]

- Furthermore, there has been discussion about the rates of discounting effects [e.g. 31,48,49], especially in the field of prevention. Effect outcomes should be discounted because the value of QALYs or health increases with time [50,51] and otherwise this value change is not taken into account in economic evaluations [52]. On the one hand, it has been argued that the same value of discounting should be used for costs and effects in order to be consistent [53], while on the other hand it has been recommended that effects should be discounted at lower rates to correct for the increasing monetary value of health over time [31,39,44]. [...]

- Additional objective measures, such as medication registration at pharmacies and data from insurance companies, and cost-diaries [59] could be included in future studies."

Other information

23) CONSORT: Registration number and name of trial registry

Dutch Trial Register NTR2168

24) CONSORT: Where the full trial protocol can be accessed, if available

Schulz DN, Kremers SP, Van Osch LA, Schneider F, Van Adrichem MJ, De Vries H. Testing a Dutch web-based tailored lifestyle program among adults: a study protocol. BMC Public Health 2011;11:108. PMID: 21324181

The study design and the different types of intervention are described in detail. However, the economic evaluation is not described in the study protocol.

25) CONSORT: Sources of funding and other support (such as supply of drugs), role of funders

"This study was funded by ZonMw, the Netherlands Organization for Health Research and Development (grant number: 120610012). The authors wish to thank the project partners at the Regional Health Authorities of the Dutch provinces of North-Brabant (GGD Brabant-Zuidoost; GGD Hart voor Brabant; and GGD West-Brabant) and Zeeland (GGD Zeeland) for their assistance in implementing the intervention and recruiting the participants. The authors wish to thank Mathieu van Adrichem, Joris Peters and Anika Thielmann for data cleaning/data entry assistance."

X26-i) Comment on ethics committee approval

x26-ii) Outline informed consent procedures

"Respondents who completed the online version of the questionnaire were invited to take part in the present study. For participation, they had to give informed consent and provide their e-mail address."

X26-iii) Safety and security procedures

X27-i) State the relation of the study team towards the system being evaluated

"Hein de Vries is scientific director of Vision2Health, a company that licenses evidence-based innovative computer-tailored health communication tools. No other authors reported any conflicts of interest."
